# The General Practitioner's Guide to Diseases and Injuries of the Eye and Eyelids

**Published:** 1884-03

**Authors:** 


### Notes on Books.

---

*The General Practitioner's Guide to Diseases and Injuries of the Eye and Eyelids.* By L. H. TOSSWILL, M.B., Pp. 147. London: J. & A. CHURCHILL, 1884.

The tendency of many authors of late years has been simply to condense or abridge the material contained in large standard works, and to bring out the result of their labours with such titles as "Guide," "Elements," "First Steps," &c.

This method has advantages for the student, no doubt; but only when it is not carried too far. When the curtailment is so great as to cause the omission of large portions of the subject there are grave objections to the plan. The author of the small work before us says in the preface that "brevity" has been his aim, so that busy practitioners, who have not time to read "more comprehensive" works, may yet be able to turn to his in the hour of need. Most men in active practice do not, however, read text-books—large or small—systematically through, but use their library for *reference* only; and for this purpose a work which embraces the whole of a subject, and not merely a fragment of it, is requisite. All the information contained in this book can readily be found, certainly in not too diffuse a form, in any of the smaller standard works, *e.g.*, those of Macnamara, Lawson, or Nettleship. The possession of this work alone would not satisfy a man with any love of his profession: he would want a more complete treatise.

Speaking generally we can say that the book is in a very readable form, and that the teaching is sound so far as it goes; but there is evidence of want of care in revision. Slight grammatical errors appear here and there, and expressions whose meaning is somewhat obscure. What are we to understand by the last part of the following sentence? "Hot belladonna or poppy fomentations are required in almost all cases of suppurative iritis, in the other varieties of iritis when attended with unusual pain, and in all except severe cases of atropism." Again, after enumerating other causes of hypopyon, the author goes on to say—"Lastly, hypopyon may be due to iritis, irrespective of suppurative inflammation."

We are told in another place that eserine should be used in suppurative corneitis only when "there seems to be no probability of iritis." Doubtless quite right in theory; but who is to decide in such cases when there is likely to be immunity from this complication?

Recourse to a specialist is frequently and rightly advised when certain operations are necessary; but are we to think that "epilation" is a manipulation requiring a specialist's skill? Yet this means of relief in cases of entropion and trichiasis is not even mentioned in the short paragraph devoted to these affections. Saemisch's operation is spoken of in two or three places. If it is necessary to mention it at all in such a work, why not say what it is, since this might be done in a very few words?

A description of the group of subjective symptoms, which should lead the practitioner to suspect the presence of "errors of refraction," ought certainly to have been inserted in any work, even when of such limited scope as this one. The estimation of such errors can, of course, only be satisfactorily accomplished by a specialist; but the practitioner should nevertheless have his earnest attention drawn to the subject, if not for the sake of his own reputation, then certainly for the benefit of his patients, many of whom would thus be spared the infliction of gallons of medicine, for intractable headaches, &c., when suitable glasses would at once effect a cure.

